# Mild KCC2 Hypofunction Causes Inconspicuous Chloride Dysregulation that Degrades Neural Coding

**DOI:** 10.3389/fncel.2015.00516

**Published:** 2016-01-29

**Authors:** Nicolas Doyon, Steven A. Prescott, Yves De Koninck

**Affiliations:** ^1^Institut Universitaire en Santé Mentale de QuébecQuébec, QC, Canada; ^2^Department of Mathematics and Statistics, Université LavalQuébec, QC, Canada; ^3^Program in Neurosciences and Mental Health, Hospital for Sick ChildrenToronto, ON, Canada; ^4^Department of Physiology, University of TorontoToronto, ON, Canada; ^5^Department of Psychiatry and Neuroscience, Université LavalQuébec, QC, Canada

**Keywords:** chloride homeostasis, chloride dynamics, GABA_A_ receptors, synaptic inhibition, disinhibition, ionic plasticity, gain control, information transfer

## Abstract

Disinhibition caused by Cl^−^ dysregulation is implicated in several neurological disorders. This form of disinhibition, which stems primarily from impaired Cl^−^ extrusion through the co-transporter KCC2, is typically identified by a depolarizing shift in GABA reversal potential (*E*_GABA_). Here we show, using computer simulations, that intracellular [Cl^−^] exhibits exaggerated fluctuations during transient Cl^−^ loads and recovers more slowly to baseline when KCC2 level is even modestly reduced. Using information theory and signal detection theory, we show that increased Cl^−^ lability and settling time degrade neural coding. Importantly, these deleterious effects manifest after less KCC2 reduction than needed to produce the gross changes in *E*_GABA_ required for detection by most experiments, which assess KCC2 function under weak Cl^−^ load conditions. By demonstrating the existence and functional consequences of “occult” Cl^−^ dysregulation, these results suggest that modest KCC2 hypofunction plays a greater role in neurological disorders than previously believed.

## Introduction

The balance of synaptic excitation and inhibition is critical for proper neural processing (Shadlen and Newsome, 1994; Wong et al., 2000; Soto-Trevino et al., 2001; Haider et al., 2006; Buzsáki et al., 2007; Vogels and Abbott, 2009). Fast inhibition, which is mediated principally by GABA_A_ receptors, depends on the magnitude and direction of Cl^−^ current (Gulledge and Stuart, 2003; De Koninck, 2007; Farrant and Kaila, 2007; Jean-Xavier et al., 2007; Doyon et al., 2011). Outward Cl^−^ current causes either frank hyperpolarization or mitigates (shunts) depolarization caused by concurrent synaptic excitation. Chloride current is typically outward (i.e., Cl^−^ flows into the neuron) because [Cl^−^]_i_ is normally maintained at a low level of 5–10 mM in mature central neurons (Staley and Proctor, 1999) rendering *E*_GABA_ at or slightly below resting membrane potential. This is achieved for the most part via the K^+^-Cl^−^ co-transporter KCC2, which (except during overt extracellular K^+^ accumulation) extrudes Cl^−^ from the cell (DeFazio et al., 2000; Payne et al., 2003; Kahle et al., 2008; Krishnan and Bazhenov, 2011). Chloride dysregulation caused by reduction of KCC2 expression or function is an important cause of disinhibition associated with several neurological and psychiatric disorders including epilepsy, chronic pain, motor spasticity, and schizophrenia (Coull et al., 2003; Jin et al., 2005; Price et al., 2005; Huberfeld et al., 2007; Hewitt et al., 2009; Boulenguez et al., 2010; Kaila and Miles, 2010; Arion and Lewis, 2011; Hyde et al., 2011; Ben-Ari et al., 2012). Throughout the text, we refer to reduced expression or function of KCC2 simply as a decrease in KCC2 level.

**Figure 1:**
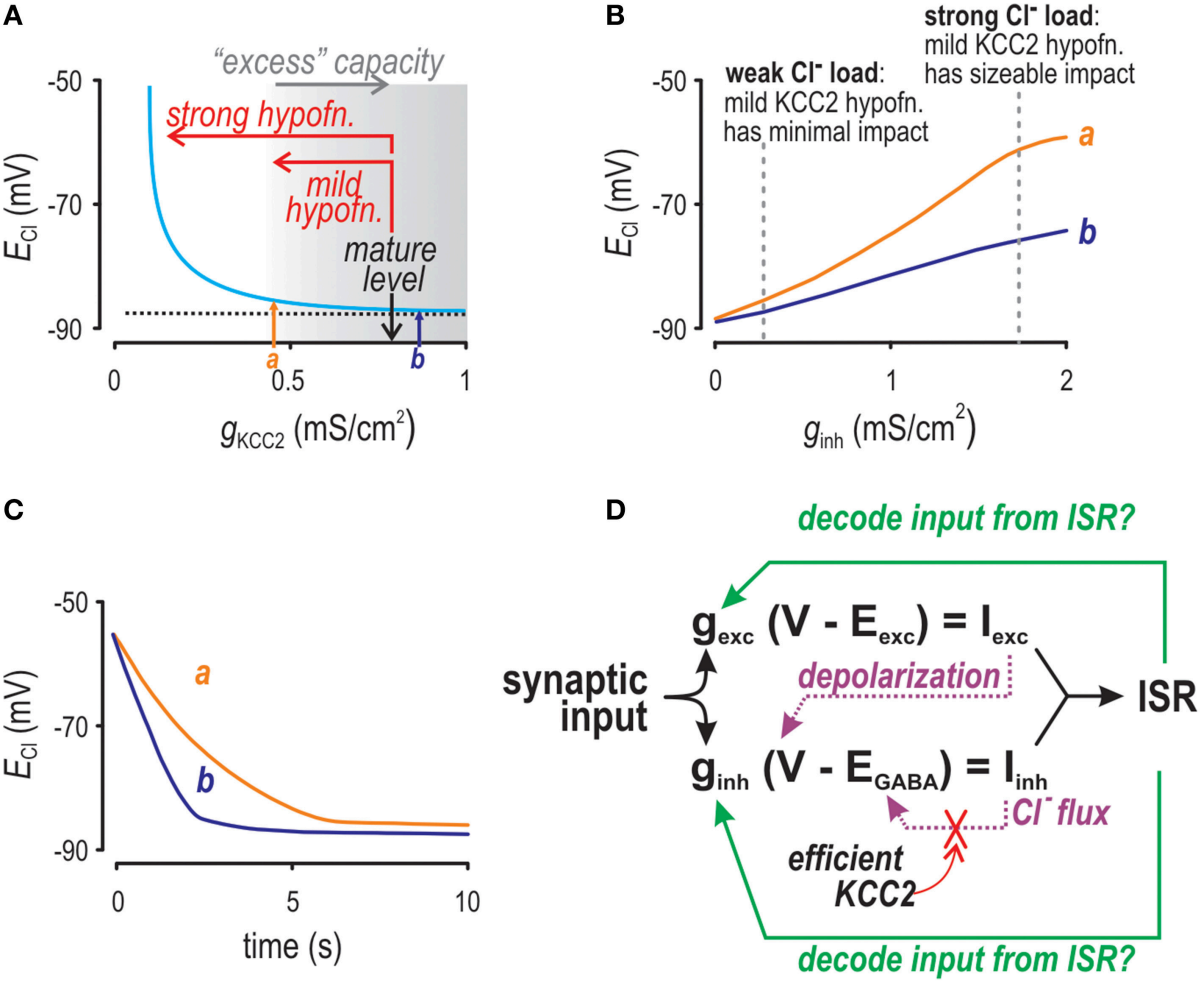
**Factors affecting Cl^−^ accumulation as investigated by computer simulation**. **(A)** *E*_Cl_ depends non-linearly on KCC2 level. When tested with weak Cl^−^ loads (as shown here), only strong KCC2 hypofunction causes an obvious depolarizing shift in *E*_Cl_. Arrows *a* and *b* indicate KCC2 levels that are both associated with normal values of *E*_Cl_ but for which differences in Cl^−^ regulation are revealed by testing in panels **(B,C)**. **(B)** As Cl^−^ load is increased, *E*_Cl_ experiences a depolarizing shift for conditions in which KCC2 level is normal (b) or modestly reduced (a), but the shift is greater in the latter case. We refer to this exaggerated shift as increased lability. **(C)** After initializing [Cl^−^]_i_ at a high level, we monitored how quickly [Cl^−^]_i_ recovers to baseline. Settling time was much slower for the modestly reduced KCC2 level (a) compared with the normal level (b). **(D)** Schematic showing the inter-relationships between factors modulating instantaneous spike rate (ISR).

But KCC2 must not only maintain [Cl^−^]_i_ under resting conditions, it must also respond to increased Cl^−^ loads that occur during synaptic inhibition. Indeed, strong GABAergic input can cause sizeable increases in [Cl^−^]_i_ (Thompson and Gahwiler, 1989; Kaila, 1994; Staley et al., 1995; Doyon et al., 2011), indicating that Cl^−^ extrusion capacity is transiently overwhelmed. Such changes have been shown to perpetuate epileptiform activity during the clonic phase of seizures (Ellender et al., 2014) and are important for the operation of rhythmic motor networks (Viemari et al., 2011). An important aspect of Cl^−^ homeostasis is the degree to which KCC2 is overwhelmed and how quickly it regains control or, in other words, how strongly and how rapidly increases in Cl^−^ are quenched by KCC2. Greater Cl^−^ extrusion capacity is needed to prevent strong Cl^−^ loads from increasing [Cl^−^]_i_ and for [Cl^−^]_i_ to recover quickly from whatever increases do occur than is needed to maintain [Cl^−^]_i_ during weak Cl^−^ loads (Figures 1A–C; Thompson and Gahwiler, 1989; Staley and Proctor, 1999; Cordero-Erausquin et al., 2005; Grob and Mouginot, 2005; Doyon et al., 2011). Indeed, during development, greater upregulation of KCC2 is required to endow the neuron with its full Cl^−^ extrusion capacity than is required for *E*_GABA_ to reach its mature value when measured under low Cl^−^ load conditions (Rivera et al., 1999; Cordero-Erausquin et al., 2005; Blaesse et al., 2006). Conversely, pathological reduction of KCC2 ought to reduce “excess” Cl^−^ extrusion capacity before manifesting an obvious change in *E*_GABA_, implying that the earliest effect of KCC2 reduction is to compromise the robustness and rapidity of Cl^−^ regulation during transient Cl^−^ loads or, in other words, to increase the lability and settling time of [Cl^−^]_i_. Indeed, collapse of inhibition during repetitive inhibitory input is accelerated in pathological conditions such as epilepsy, stress disorders, and chronic pain (Jin et al., 2005; Hewitt et al., 2009; Ferrini et al., 2013) but the precise dependence on KCC2 level, especially with respect to settling time, has not been rigorously explored. The necessary starting point of the current study was, therefore, to clarify how the lability and settling time of [Cl^−^]_i_ depend on neuronal factors, most notably KCC2 level.

Disinhibition will naturally cause a net increase in excitability within affected neurons and neural networks. However, not all disinhibitory mechanisms will manifest under the same conditions or have precisely the same impact on neural function. For instance, disinhibition caused by KCC2 hypofunction, unlike that caused by reduced GABAergic transmission, is activity dependent (i.e., predominant when KCC2 function is transiently overwhelmed by increased GABAergic transmission). We hypothesized that the increased Cl^−^ lability and settling time caused by modest KCC2 hypofunction compromise neural coding under physiologically realistic conditions in which GABAergic input (and, by extension, Cl^−^ load) fluctuate (Destexhe and Paré, 1999; Sernagor et al., 2003). To test this, we conducted simulations in a neuron model in which [Cl^−^]_i_ and *E*_GABA_ are dynamically updated, and we used information theory, signal detection theory, and other metrics to quantify the impact of KCC2 function on neural coding. Our results demonstrate that neural coding can be substantially degraded in the absence of conspicuous changes in the resting value of *E*_GABA_. These results suggest that modest KCC2 hypofunction—subtle enough to go undetected by experiments that do test elevated Cl^−^ loads—can have important consequences for neural coding. By extension, modest KCC2 hypofunction could have more widespread consequences than previously believed.

## Materials and methods

### Extended morris-lecar model

We used a single-compartment, conductance-based Morris-Lecar model (Morris and Lecar, 1981). The basic equations describing the dynamic behavior of membrane potential (*V*) and recovery variable (*w*) were:

$$\begin{array}{l} \frac{dV}{dt}=-\frac{1}{Cap}(\bar{g_{Na}}\left(V-E_{Na}\right)0.5\left(1+\text{tanh}\left(\frac{V-v_{1}}{v_{2}}\right)\right)\\ \;+\bar{g_{k}}w\left(V-E_{K}\right)+g_{inh}\left(V-E_{GABA}\right)+g_{exc}\left(V-E_{exc}\right)\\ \;+g_{L}\left(V-E_{L}\right)+I_{ahp}+I_{Ca}),\\ \frac{dw}{dt}=\frac{\varphi \left(0.5\left(1+\text{tanh}\left(\frac{V-v_{3}}{v_{4}}\right)\right)-w\right)}{\text{sech}\left(\frac{V-v_{3}}{2v_{4}}\right)}, \end{array}$$

where *Cap* = 2 μF/cm^2^, φ = 0.25, *v*_1_ = −1.2 mV, *v*_2_ = 18 mV, *v*_3_ = −9 mV, *v*_4_ = 10 mV (Prescott et al., 2008). The value of *E*_GABA_ (in mV) was given by the Goldman-Hodgkin-Katz equation:

$$\begin{array}{lll} E_{GABA} & = & 1000\left(\frac{RT}{F}\right)\text{ln}\left(\frac{{4\left[{Cl}^{-}\right]}_{i}+{\left[{{HCO}_{3}}^{-}\right]}_{i}}{4{\left[{Cl}^{-}\right]}_{o}+{\left[{{HCO}_{3}}^{-}\right]}_{o}}\right), \end{array}$$

where *T* = 310 K stands for absolute temperature, *R* = 8.3 J/(K·mol) for the perfect gas constant, and *F* = 96 485 C/mole for the Faraday constant. Except for [Cl^−^]_i_ and [Ca^2+^]_i_, other ionic concentrations were treated as constant: *E*_Na_ = 45 mV and *E*_K_ = −85 or −95 mV (Hille, 2001). The intra and extra cellular bicarbonate concentrations were set to [${\text{HCO}}_{3}^{-}$]_i_ = 11.8 mM and [${\text{HCO}}_{3}^{-}$]_*o*_ = 25 mM (Staley and Proctor, 1999). Leak reversal potential (*E*_L_) was set at −70 mV (Hille, 2001), the reversal potential of excitatory synapses (*E*_*exc*_) was set to 0 mV (Jahr and Stevens, 1993) and [Cl^−^]_o_ was fixed at 120 mM. Chloride reversal potential (*E*_Cl_) in units of mV was computed using Nernst equation:

$$\begin{array}{l} E_{Cl}=1000\left(\frac{RT}{F}\right)\text{ln}\left(\frac{{\left[{Cl}^{-}\right]}_{i}}{{\left[{Cl}^{-}\right]}_{o}}\right). \end{array}$$

The Cl^−^ efflux through KCC2 was given by the linear approximation *g*_*KCC*2_(*E*_*K*_*-E*_*Cl*_) which is a simplification of more comprehensive models (Williams et al., 1999; Williams and Payne, 2004). Intracellular [Cl^−^] was updated according to:

$$\begin{array}{l} \frac{d{\left[{Cl}^{-}\right]}_{i}}{dt}=SAV\frac{g_{KCC2}\left(E_{Cl}-E_{K}\right)-{xg}_{inh}\left(V_{m}-E_{Cl}\right)}{F}, \end{array}$$

where *SAV* is the is the surface area to volume ratio of the cell taken to be surface the surface area to volume ratio of a sphere so that *SAV* = 10^−1^ *s*/*r* where *s* = 3 for a sphere and *r* = 6 μm (Ratté and Prescott, 2011) unless otherwise indicated. The variable *x* describes the fraction of the GABA_A_ mediated current that is due to the flux of Cl^−^ ions (Ratté and Prescott, 2011) namely:

$$\begin{array}{l} x=\frac{V-E_{Cl}}{V-E_{GABA}}. \end{array}$$

We also modeled changes in [Ca^2+^]_i_ according to:

$$\begin{array}{l} \frac{d{\left[{Ca}^{2+}\right]}_{i}}{dt}=SAV\frac{I_{Ca}}{2\cdot F}+\frac{{\left[{Ca}^{2+}\right]}_{i,rest}-{\left[{Ca}^{2+}\right]}_{i}}{\tau_{Ca}}, \end{array}$$

as described in De Schutter and Somlen (1998) with [Ca^2+^]${}_{i,rest}=10^{-4}$ mM (Collins et al., 1991; Nakajima et al., 1993) and τ_*Ca*_ = 50 ms (Traub and Llinas, 1977). The calcium current, *I*_Ca_ was given in μA/cm^2^ by the equation:

$$\begin{array}{l} I_{Ca}=\bar{g_{Ca}}\left(V-E_{Ca}\right)\frac{1}{1+\exp\left[-\left(V-V_{lt}\right)∕k_{l}\right]}, \end{array}$$

with $\bar{g_{\text{Ca}}}=0.5$ mS/cm^2^, *V*_*lt*_ = −5 mV and *k*_*l*_ = 5 mV (Benison et al., 2001). The value of *E*_ca_ was computed using [Ca^2+^]_*o*_ = 5·10^−3^ M (Jahr and Stevens, 1993). Calcium concentration was then used to determine the kinetics of Ca^2+^ activated K^+^ channels limiting high frequency spiking. The dynamics of those channels were described by the following equations:

$$\begin{array}{l} I_{ahp}=\bar{g_{ahp}}m_{ahp}\left(V-E_{K}\right), \end{array}$$

with $\bar{g_{ahp}}=1$ mS/cm^2^ (Destexhe and Paré, 1999) and

$$\begin{array}{l} m_{ahp}=\frac{{\left[{Ca}^{2+}\right]}_{i}^{2}}{{K_{Ca}}^{2}+{\left[Ca^{2+}\right]}_{i}^{2}} \end{array}$$

with $K_{\text{Ca}}=10^{-3}$ mM (Moczydlowski and Latorre, 1983). Model parameters related to Cl^−^ dynamics were validated by comparing the Cl^−^ extrusion rate when [Cl^−^]_i_ = 15 mM to the half maximal extrusion rate reported in Staley et al. (1995). All simulations were performed with MATLAB software and equations were integrated using forward Euler method.

### Ornstein-uhlenbeck process

**Figure 2:**
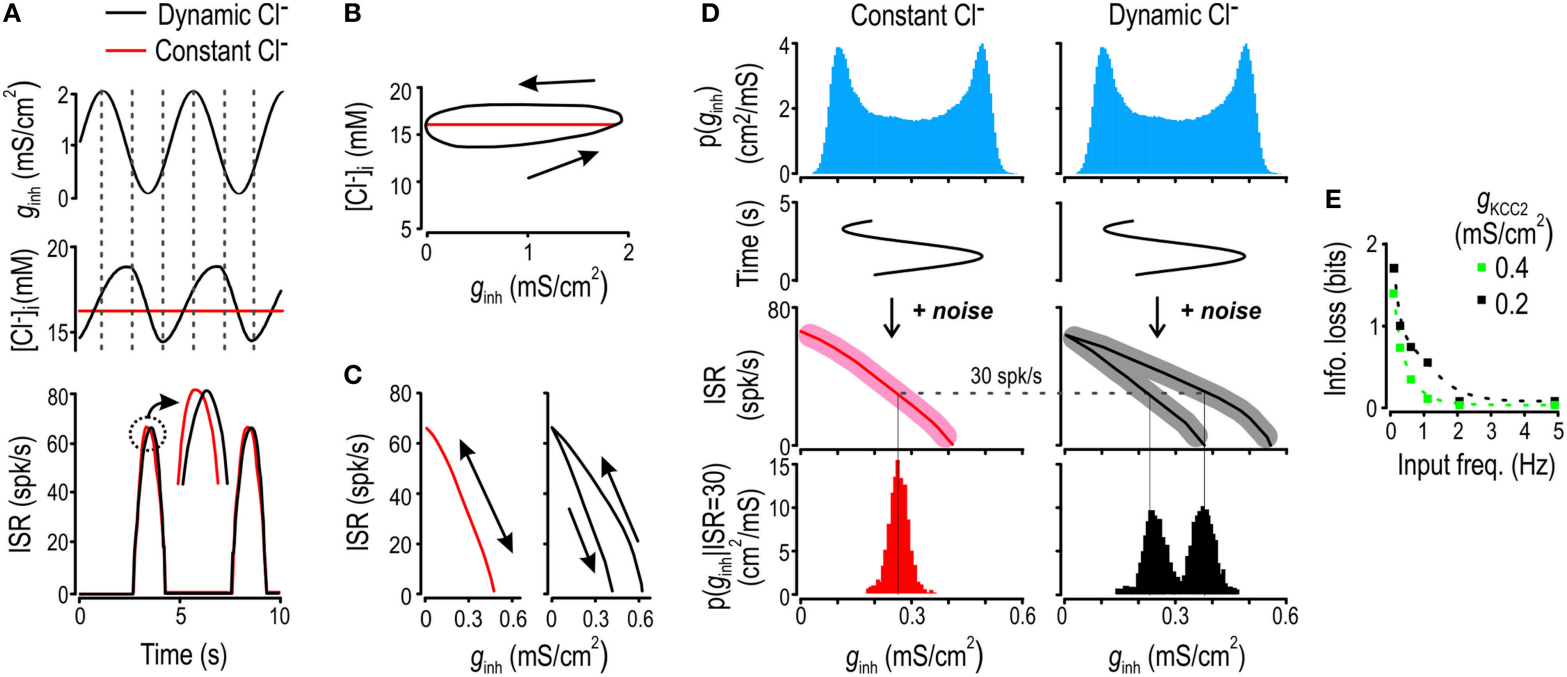
**Chloride fluctuations arise from and impair encoding of slow inhibitory inputs**. **(A)** Model neuron was given *g*_*inh*_ stimulus oscillating at 0.2 Hz (*top*) and the resulting fluctuations in [Cl^−^]_i_ (*middle*) and ISR (*bottom*) were monitored under dynamic and static [Cl^−^]_i_ conditions (*black* and *red* respectively). Chloride concentration **(B)** and ISR **(C)** are displayed as functions of *g*_*inh*_. The model with static [Cl^−^]_i_ displays a single branch whereas the model with dynamic [Cl^−^]_i_ shows separate branches for the ascending and descending phases of the input. **(D)** Model was given 0.2 Hz *g*_*inh*_ sinusoidal input with superimposed noise. Blue histogram shows the noiseless input distribution. For dynamic and static [Cl^−^]_i_ conditions, we computed the input-output curves without added noise (*full lines*) and with added noise (*shaded area*). From these, we computed the conditional distributions of *g*_*inh*_ for ISR = 30 Hz for scenario of static and dynamic [Cl^−^]_i_ (*red and black histograms*). **(E)** By computing the entropy of these conditional distributions and averaging over all possible values of ISR, we obtained the fraction of information transfer lost due to Cl^−^ lability, which is plotted as a function of input frequency for modest (*green*) and strong (*black*) KCC2 downregulation.

**Figure 3:**
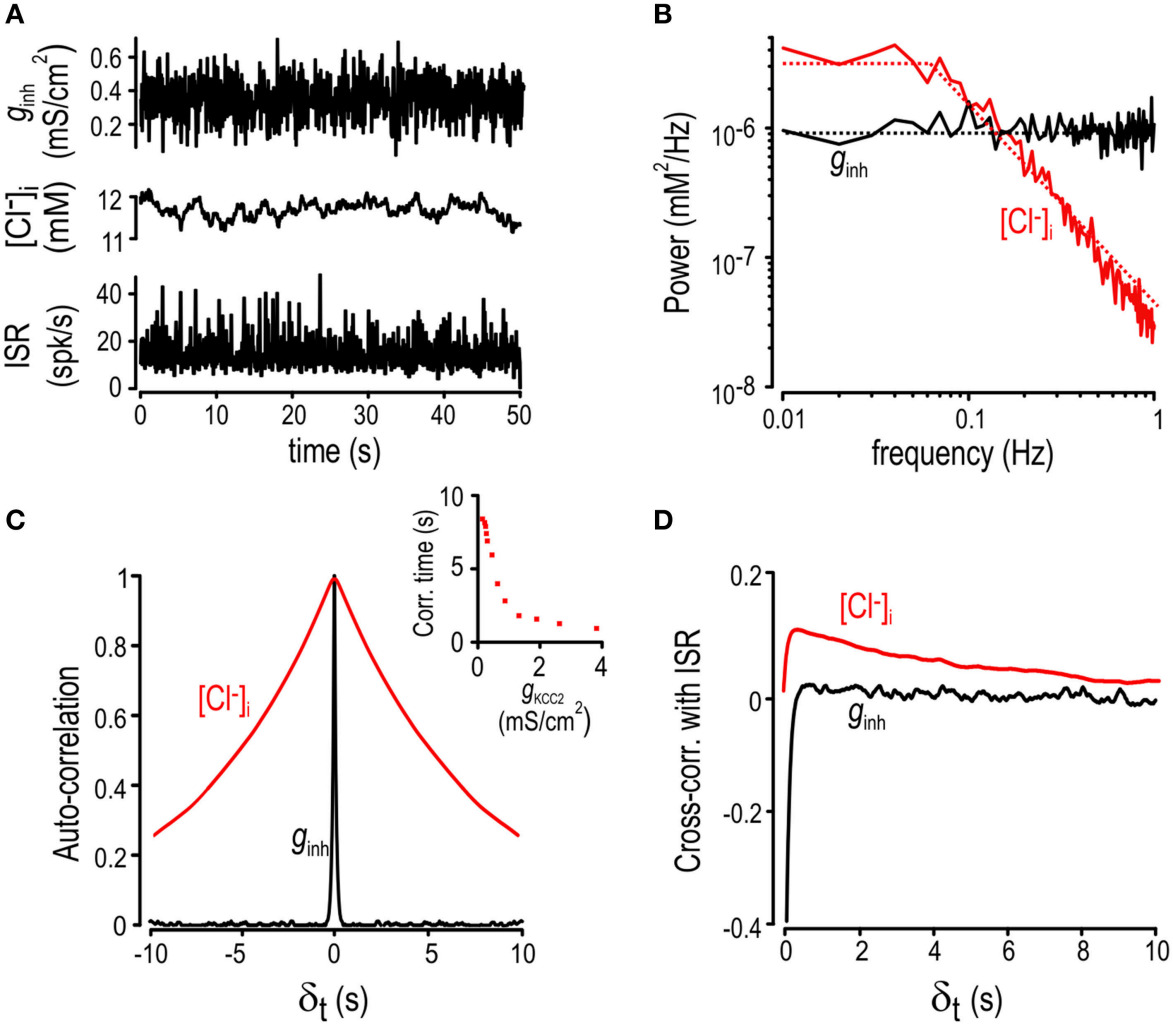
**Chloride fluctuations reflect low-pass filtering of *g*_*inh*_**. **(A)** Inhibitory input was generated with an Ornstein-Uhlenbeck process (*top*) while excitatory conductance was kept constant. Fluctuations in [Cl^−^]_i_ (*middle*) and ISR (*bottom*). **(B)** Power spectra of the inhibitory input (*black*) and the [Cl^−^]_i_ fluctuations (*red*). Smoothed values are also displayed as dotted lines. **(C)** Autocorrelation graphs for [Cl^−^]_i_ (*red*) and *g*_*inh*_ (*black*). Inset shows autocorrelation time of [Cl^−^]_i_ fluctuations determined from simulations with different KCC2 levels. **(D)** Cross-correlation between the ISR and *g*_*inh*_ (*black*) as well as between the ISR and [Cl^−^]_i_ (*red*).

In simulations of **Figures 3, 7** the signal was generated through an Ornstein-Uhlenbeck process (Uhlenbeck and Ornstein, 1930). The time evolution of the signal strength *x*_*t*_ is described by the following equation

$$\begin{array}{l} dx_{t}=\theta \left(\mu -x_{t}\right)dt+\sigma dW_{t} \end{array}$$

where *W*_*t*_ represent a Wiener process, μ is the mean of the process equal to 0. In simulations of Figure 2, the same Ornstein-Uhlenbeck process was used to generate noise added to a sinusoidal signal. For the simulations in Figure 3 we used the following parameters: θ = 11 s^−1^ and $\sigma =\sqrt{\frac{2.2\;mS∕{\text{cm}}^{2}}{\text{s}}}$. The simulations in Figure **7** were performed with different values of μ and σ to adjust the mean value and standard deviation of the process in accordance to the descriptions in the figure section and the figure legends.

### Mutual information

Mutual information between two variables *X* and *Y* was computed using Shannon's mutual information formula (Shannon, 1949):

I(X,Y)=∬[p(x,y)(log(p(x,y))−log(p(x)p(y))]dxdy.

Alternatively, the mutual information between *X* and *Y* is given by I(*X*,*Y*) = H(*X*) − H(*X*|*Y*) where H(*X*) stands for the Shannon's entropy of the variable *X* which is given by:

$$\begin{array}{l} H\left(X\right)=-\int p\left(x\right)\log\left(p\left(x\right)\right)dx \end{array}$$

and *X*|*Y* is the conditional distribution of *X* knowing *Y*. Computations were performed using distributions discretized into 100 bins.

### Power spectrum and transfer functions

Power spectrums in Figure 3 were computed using fast-Fourier-transform functions in MATLAB. Autocorrelation of a quantity *X* at delay time δ was computed according to the formula:

$$\frac{\int_{0}^{L-\delta }X(t)X(t+\delta )dt-{\left(\int_{0}^{L-\delta }X(t)dt\right)}^{2}}{\int_{0}^{L-\delta }X^{2}(t)dt-{\left(\int_{0}^{L-\delta }X(t)dt\right)}^{2}},$$

where *L* is the time length of the simulation. Cross-correlation between two time vectors *X* and Y was computed according to the equation:

$$\frac{\int_{0}^{L-\delta }X(t)Y(t+\delta )dt-\left(\int_{0}^{L-\delta }X\left(t\right)dt\right)\left(\int_{0}^{L-\delta }Y(t)dt\right)}{\sqrt{\int_{0}^{L-\delta }X^{2}(t)dt-{\left(\int_{0}^{L-\delta }X(t)dt\right)}^{2}}\sqrt{\int_{0}^{L-\delta }Y^{2}(t)dt-{\left(\int_{0}^{L-\delta }Y(t)dt\right)}^{2}}}.$$

To compute the transfer functions in Figure 3, we submitted the model neuron to a series of sinusoidal inhibitory inputs with *g*_*inh*_ oscillating between 0 and 2 mS/cm^2^ at frequencies between 0.01 and 1 Hz. For each input frequency, we computed the amplitude of the Cl^−^ fluctuation [Δ [Cl^−^]_i_ = max([Cl^−^]_i_) – min([Cl^−^]_i_)] after initial convergence as well as phase delay [[*Time*_max(*Cl*)_ - *Time*_max(*ginh*)_]/*Per*] where *Per* is the period of input oscillations.

### Discriminability and ROC analysis

For the ROC analysis performed in **Figure 7**, the model neuron was submitted to excitatory and inhibitory inputs generated with an Ornstein-Uhlenbeck process as described above. For each KCC2 level and excitation-inhibition ratio, we measured ISR histograms. For thresholds running from the minimum ISR obtained to the maximum ISR value, we computed the proportion of ISR from input with *g*_*inh*_/*g*_*exc*_ = 1 smaller than this threshold and the proportion of ISR from input with *g*_*inh*_/*g*_*exc*_ = 1 smaller than this threshold. When plotted against each other, these two probabilities yielded the ROC curves.

## Results

### Overview of Cl^−^ dynamics and their impact on neural coding

In healthy adult central neurons, Cl^−^ influx via GABA_A_ receptors is counterbalanced by Cl^−^ efflux via KCC2 and the equilibrium value of [Cl^−^]_i_ depends on the strength of each process. Intracellular [Cl^−^] can thus change with time if GABA_A_ input (i.e., conductance) and/or Cl^−^ driving force vary, where changes in the latter can arise from variations in *E*_GABA_ or by membrane depolarization caused by concurrent synaptic excitation (Figure 1D; Thompson and Gahwiler, 1989; Doyon et al., 2011). Relationships identified in Figure 1D raise important points. First, inhibitory current (*I*_*inh*_) and *E*_GABA_ form a negative feedback loop, the gain of which is inversely related to the strength of KCC2 activity. The negative nature of the feedback is liable to convey a counterproductive form of plasticity in which inhibition weakens under precisely the conditions in which it is most needed. Second, ISR is assumed to reflect the ratio of excitatory and inhibitory input, implying that we can infer (decode) that input from ISR; however, that assumption is violated if driving forces vary. Since stability of the Cl^−^ driving force depends on the feedback loop between *I*_*inh*_ and *E*_GABA_, the gain of which depends on KCC2 (see Figure 1D), it stands to reason that ability to decode synaptic input from ISR is dependent on KCC2. Having established these relationships qualitatively, we sought to quantify their impact on neural coding.

To quantify the impact of Cl^−^ dynamics on the encoding of inhibitory input, we first simulated a 0.2 Hz sinusoidal inhibitory conductance *g*_*inh*_ (Figure 2A *top*) in a model in which [Cl^−^]_i_ fluctuates freely for comparison with a model in which [Cl^−^]_i_ is artificially fixed. To isolate the effects of Cl^−^ lability, the fixed [Cl^−^]_i_ value was chosen to equal the mean value of [Cl^−^]_i_ under dynamic conditions (Figure 2A *middle*). When plotted against time, ISR exhibited a seemingly trivial phase difference between the two models (Figure 2A *bottom*). However, the elliptical orbit revealed by plotting [Cl^−^]_i_ against *g*_*inh*_ shows that [Cl^−^]_i_ differs between the ascending and descending phases of the stimulus (Figure 2B)—this results from changes in [Cl^−^]_i_ lagging behind changes in the input. The efficacy of inhibition depends on Cl^−^ current (see above), which means that time-dependent variation in [Cl^−^]_i_, by affecting that current through *E*_GABA_ and driving force, will produce time-dependent modulation of ISR. Consequently, whereas ISR is a well-defined function of *g*_*inh*_ in the model with static [Cl^−^]_i_, ISR becomes ambiguously related to *g*_*inh*_ in the model with dynamic [Cl^−^]_i_ (Figure 2C, red and black curves, respectively).

The slow Cl^−^ fluctuations described above evidently preclude the value of *g*_*inh*_ at time *t* from being unambiguously decoded from ISR. To quantify this effect, we added noise to a sinusoidally varying *g*_*inh*_ “signal” and measured the amount of information transmitted by ISR on that signal in dynamic vs. static [Cl^−^]_i_ conditions. Information transfer was calculated based on conditional entropy, which, in a very generic way, quantifies how much more confidently one can predict the input when one knows the output. The relationship between ISR and *g*_*inh*_ having two branches for dynamic [Cl^−^]_i_ conditions results in greater conditional entropy, which equates with ISR providing less information about *g*_*inh*_, as illustrated in Figure 2D (note the broader and bimodal conditional distribution of *g*_*inh*_ in the case of fluctuating [Cl^−^]_i_) for an arbitrarily chosen response of 30 spikes/s to *g*_*inh*_ sinusoidally modulated at 0.2 Hz. The more broadly those branches are separated (Supplementary Figure 1), the greater the conditional entropy and the less information ISR provides about *g*_*inh*_. Re-testing with different sinusoidal *g*_*inh*_ frequencies shows that the loss of information attributable to dynamic [Cl^−^]_i_ was highest for the lowest frequencies tested and became negligible for frequencies >1 Hz (Figure 2E). These results show that lability of [Cl^−^]_i_ occurs to the detriment of information transmission when the cell experiences relatively slow variation in inhibitory input.

### Factors affecting the amplitude and kinetics of Cl^−^ fluctuations

The observation that slower inputs drive larger fluctuations (Supplementary Figure 1) suggests that [Cl^−^]_i_ is a low-pass filtered reflection of inhibitory synaptic input. To characterize that filtering, we stimulated the model neuron with noisy inhibitory input that accurately recapitulates background synaptic activity (Destexhe et al., 2001). As expected, fluctuations in [Cl^−^]_i_ were sluggish compared with the rapid changes in *g*_*inh*_ and ISR (Figure 3A). Indeed, the power spectrum for *g*_*inh*_ is flat between 0.01 and 10 Hz whereas the one for [Cl^−^]_i_ falls off beyond 0.1 Hz, consistent with low-pass filtering (Figure 3B). The slow nature of Cl^−^ fluctuations led us to postulate that [Cl^−^]_i_ at any given time should reflect past values of [Cl^−^]_i_ as well as predict its future values. This was tested by computing the auto-correlation function for both [Cl^−^]_i_ and *g*_*inh*_ (Figure 3C). Results show that *g*_*inh*_ fluctuations with an autocorrelation time of 84 ms yielded fluctuations in [Cl^−^]_i_ with a much longer autocorrelation time of 8 s. Consequently, [Cl^−^]_i_ at a given time is a good predictor of what [Cl^−^]_i_ will be 5–10 s later whereas *g*_*inh*_ is forgotten within milliseconds. The inset in Figure 3C shows that [Cl^−^]_i_ autocorrelation time is inversely related to KCC2 level, indicating that KCC2 downregulation results in slower Cl^−^ fluctuations (see below). Since [Cl^−^]_i_ affects ISR, it follows that [Cl^−^]_i_ at time *t* should be predictive of both ISR at that time *t* as well as ISR a few seconds into the future, whereas this should not be the case for *g*_*inh*_, given its short autocorrelation time. To test this, we measured the cross-correlation between [Cl^−^]_i_ and ISR as well as between *g*_*inh*_ and ISR. As predicted, cross-correlation between [Cl^−^]_i_ and ISR remained positive for a few seconds whereas *g*_*inh*_ was forgotten within milliseconds (Figure 3D).

**Figure 4:**
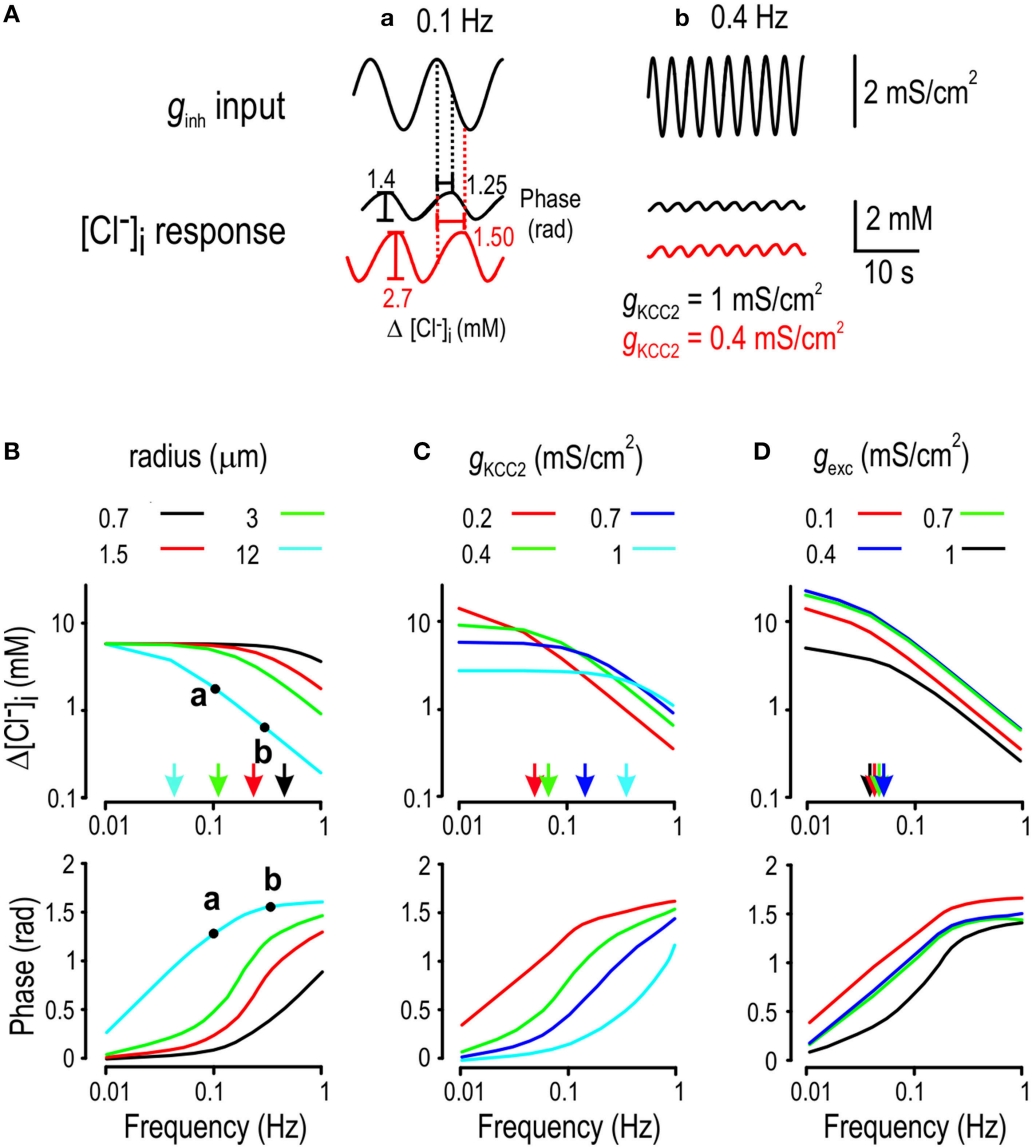
**Determinants of Cl^−^ filtering properties**. **(A)** Cartoon explaining how transfer functions were computed for normal KCC2 level (*black*) and a modestly reduced KCC2 level (*red*). **(B–D)** We submitted the model to sinusoidal inhibitory input of different frequencies and measured the amplitude (*top*) and phase (*bottom*) of the [Cl^−^]_i_ response. Arrows in panels **(B–D)** indicate corner frequencies. **(B)** Chloride concentration more accurately tracks higher frequency inputs in smaller cells. **(C)** Chloride fluctuations become larger but less capable of tracking high frequency inputs (i.e., Cl^−^ fluctuations become slower) as KCC level is reduced. **(D)** Sinusoidally modulated excitatory input (with constant inhibition) also drives Cl^−^ fluctuations by affecting the Cl^−^ driving force.

The above data confirm that Cl^−^ fluctuations reflect low-pass filtering of *g*_*inh*_ fluctuations, the details of which presumably depend on a variety of post-synaptic factors that influence the balance of Cl^−^ influx and efflux. To investigate those factors, we measured the transfer function by submitting the model neuron to sinusoidal *g*_*inh*_ of different frequencies but equal amplitude. For each input frequency, we computed the amplitude of [Cl^−^]_i_ fluctuations as well as the phase delay between fluctuations in *g*_*inh*_ and [Cl^−^]_i_ under a variety of conditions (Figure 4A). We hypothesized that cell radius would be an important determinant of filtering given its effects on the surface area-to-volume (SAV) ratio. As expected, the corner frequency of the Cl^−^ response decreased as the SAV ratio was reduced by increasing radius (Figure 4B *top*). We also found that the corner frequency was significantly affected by KCC2 level (Figure 4C *top*). Unlike varying radius, reducing KCC2 allowed for larger [Cl^−^]_i_ fluctuations at low input frequencies (Figure 4C *top*). The magnitude of excitatory input *g*_*exc*_ also influenced the magnitude of [Cl^−^]_i_ fluctuations (because depolarization increases Cl^−^ driving force; see Figure 1D), but it had little effect on the corner frequency (Figure 4D *top*). Under all conditions (Figures 4B–D *bottom*), phase delay was near zero for very low frequency inputs (indicating that [Cl^−^]_i_ tracks slow changes in input) but increased toward π/2 as input frequency was increased (indicating that [Cl^−^]_i_ lags behind faster changes in input). These results highlight that the amplitude and kinetics of Cl^−^ fluctuations are likely to differ quantitatively between different cells, or even between different subcellular compartments. One can thus predict, for example, that the axon hillock, with its small diameter, low KCC2 level (Stafstrom, 2009; Bender and Trussell, 2012) and dense GABAergic innervation will exhibit large time dependent Cl^−^ fluctuations in comparison with the soma.

The fact that decreasing KCC2 function yielded both increasing maximum [Cl^−^]_i_ fluctuation amplitude and decreasing corner frequency reflects a combination of *weak* and *slow* Cl^−^ regulation, or, in other words, increased Cl^−^ lability and settling time. It follows that an abrupt but sustained increase in *g*_*inh*_ will elicit a large change in [Cl^−^]_i_, but [Cl^−^]_i_ will be slow to reach its new steady-state. Since ISR depends on the present values of *g*_*inh*_ and [Cl^−^]_i_, and since the present value of [Cl^−^]_i_ depends on past values of *g*_*inh*_, (until [Cl^−^]_i_ converges to steady-state), ISR will depend on both present and *past* values of *g*_*inh*_. This history-dependence, or ionic memory, will naturally compromise decoding of the present value of *g*_*inh*_ from ISR, as demonstrated below.

### Impact of increased Cl^−^ lability and settling time on information transfer

**Figure 5:**
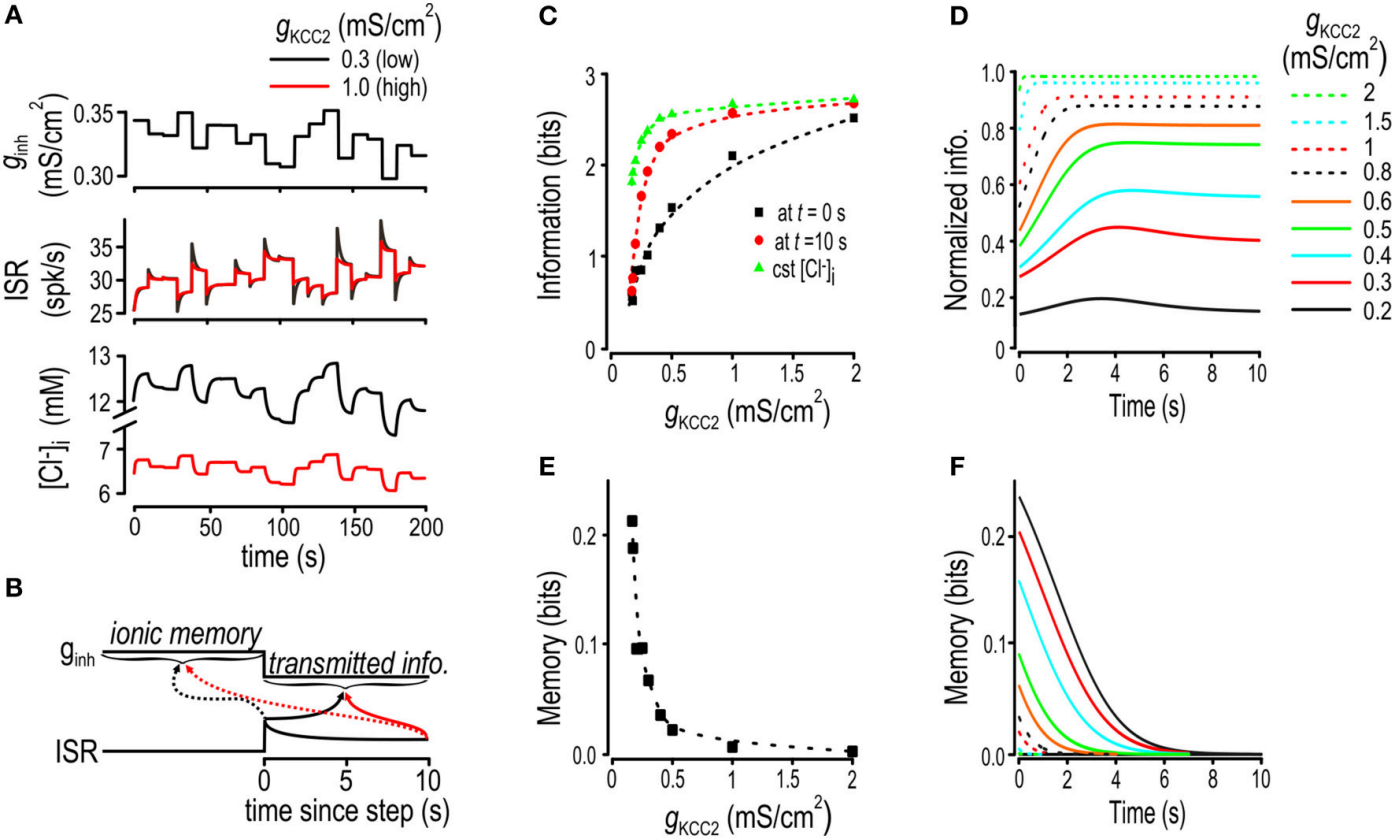
**Effects of increased Cl^−^ lability and settling time on encoding of inhibitory input**. **(A)** In these simulations, *g*_*exc*_ and *g*_*KCC*2_ were fixed and *g*_*inh*_ was abruptly changed at 10 s intervals to values chosen from a Gaussian distribution. Sample traces are shown for *g*_*inh*_, [Cl^−^]_i_ and ISR for normal and reduced KCC2 levels. **(B)** Schematic shows how ionic memory and transmitted information were calculated at different times after step changes in *g*_*inh*_. **(C)** Transmitted information for various KCC2 levels calculated immediately after the step change in *g*_*inh*_, (*black*), calculated 10 s after the step change (*red*), and calculated under static [Cl^−^]_i_ conditions (*green*). See Supplementary Figure 2 for how parameters for static [Cl^−^]_i_ condition simulations were chosen. **(D)** Transmitted information as a function of time elapsed since the last step change in *g*_*inh*_. Transmitted information is normalized by the amount of information transmitted under static [Cl^−^]_i_ conditions, with each curve representing a different KCC2 level. **(E)** Ionic memory calculated immediately after the step change in *g*_*inh*_ plotted against KCC2 level. **(F)** Ionic memory is plotted as a function of time elapsed since the step change in *g*_*inh*_ for a range of *g*_*KCC*2_ values.

To quantify the impact of Cl^−^ dynamics on information transfer, we simulated abrupt step-changes in *g*_*inh*_ applied at 10 s intervals where each value of *g*_*inh*_ was chosen from a Gaussian distribution (Figure 5A). This was repeated for different levels of KCC2 but, for each KCC2 level, we adjusted the mean of the distribution from which *g*_*inh*_ was chosen such that combinations of *g*_*KCC*2_ and mean *g*_*inh*_ yielded equivalent inhibition across all KCC2 levels (i.e., reduction of ISR from 80 spikes/s without inhibition to 30 spikes/s with inhibition; see Supplementary Figure 2 for the relationship between mean *g*_*inh*_, mean [Cl^−^]_i_ and *g*_*KCC*2_). By choosing this paradigm, we tested how KCC2 hypofunction affects the robustness and kinetics of Cl^−^ regulation without affecting the mean ISR by unbalancing excitation and inhibition. This is important because disinhibition caused by an arbitrarily applied shift in *E*_GABA_ can, depending on the degree of change, be offset by an increase in *g*_*inh*_ (Prescott et al., 2006; Knabl et al., 2008; Asiedu et al., 2010; Doyon et al., 2011). Critically, such compensation will not offset *weak* and *slow* Cl^−^ regulation resulting from KCC2 hypofunction and instead, would tend to exacerbate such changes. Indeed, our simulations reveal a tendency for ISR to overshoot and converge slowly in response to abrupt change in *g*_*inh*_ (Figure 5A).

We computed mutual information between ISR (at different time points after the step change in *g*_*inh*_) and the present and previous value of *g*_*inh*_ using the approach introduced in Figure 2D. This was repeated for multiple KCC2 levels. In effect, we asked how well *g*_*inh*_ can be decoded from ISR. The time-dependent changes in ISR, which parallel the time-dependent changes in [Cl^−^]_i_ (see Figure 5A), tend to impair that decoding. Our analysis quantifies the impairment as a function of KCC2 level; later, we will address how ionic fluctuations affect other aspects of neural coding. Here, we refer to information available from ISR about the present value of *g*_*inh*_ as *transmitted information* and to information available about the previous value of *g*_*inh*_ as *ionic memory* (Figure 5B). We compared transmitted information calculated for dynamic Cl^−^ conditions against transmitted information calculated for static Cl^−^ conditions; this does not imply that constant [Cl^−^]_i_ is necessarily optimal but, instead, serves simply to isolate the effects of dynamic changes in [Cl^−^]_i_.

Figure 5C shows transmitted information calculated at times immediately and 10 s after the step change in *g*_*inh*_ and plotted against KCC2 level. For comparison, transmitted information was also calculated for static [Cl^−^]_i_ conditions, with [Cl^−^]_i_ set to the time-averaged value associated with each KCC2 level (see Supplementary Figure 2). As predicted, less information was transmitted when KCC2 was reduced, but whereas information loss measured after 10 s remained small until large reductions in KCC2, information loss immediately after the change in *g*_*inh*_ was evident even for small reductions in KCC2 (Figures 5C,D). This differential loss of information pinpoints the effects of the slow equilibration of [Cl^−^]_i_—increased settling time—which is also evident by plotting transmitted information as a function of time elapsed since the step change in *g*_*inh*_ normalized to transmitted information with constant [Cl^−^]_i_ (Figure 5D). Patterns of transmitted information (Figures 5C,D) are inversely related to patterns of ionic memory (Figures 5E,F).

These results show that mild KCC2 hypofunction impairs information transfer on both short and long time scales. On short time scales, information loss is related to ionic memory, which is a direct consequence of *slow* Cl^−^ regulation (i.e., long settling times). On longer time scales, information loss is due to instability of the [Cl^−^]_i_ steady-state, which is a direct consequence of *weak* Cl^−^ regulation (i.e., increased lability). The slowing and weakening of Cl^−^ regulation are obviously linked—[Cl^−^]_i_ would not drift toward new steady-state values if steady-state was static—but our results highlight that the effects of slow Cl^−^ settling time are more readily manifested in terms of information loss. Based on our testing paradigm, we can also conclude that these effects are not prevented by compensating for reduced Cl^−^ driving force by increasing the level of GABA_A_ conductance.

### Anionic memory and encoding of excitatory input

**Figure 6:**
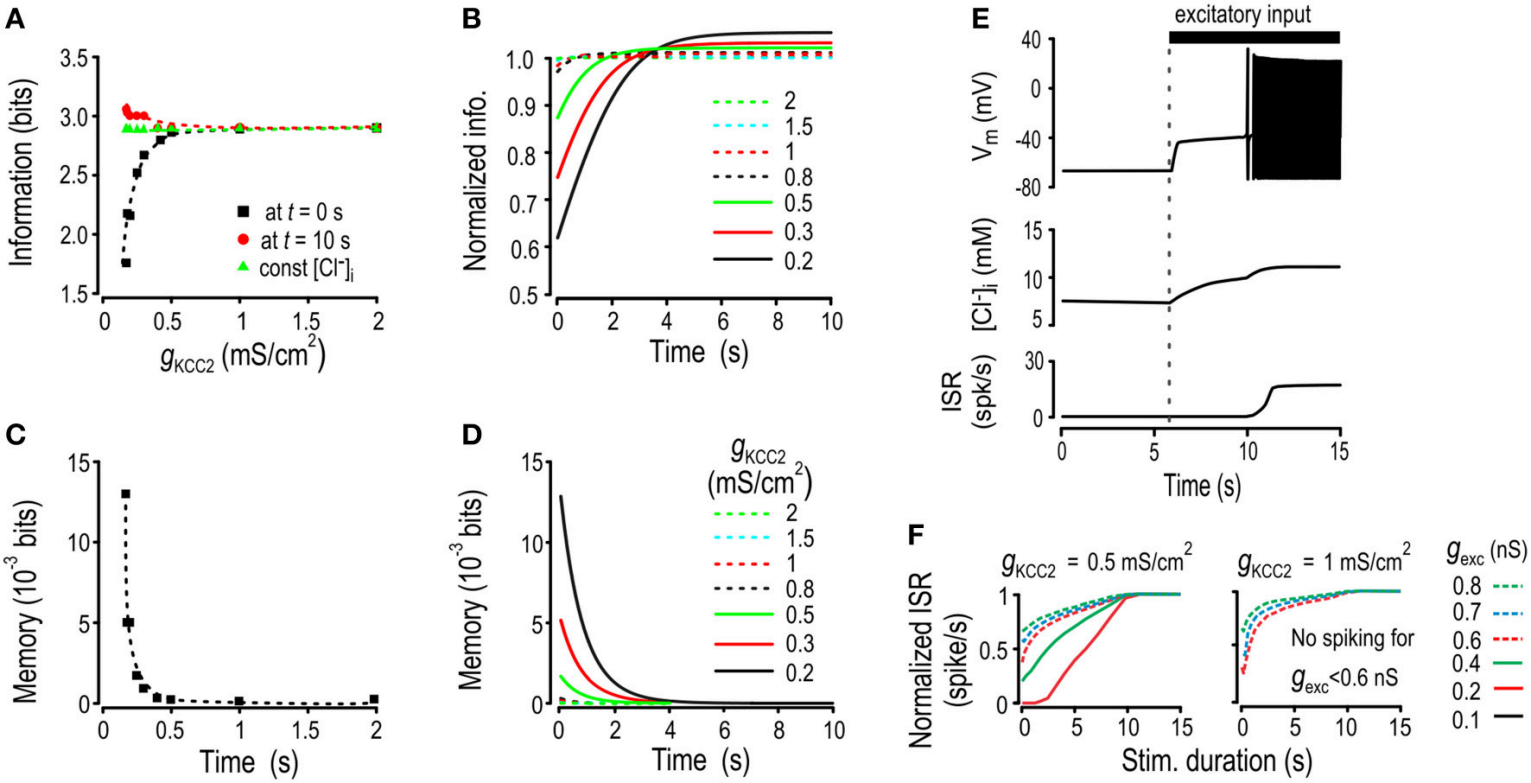
**Effects of increased Cl^−^ lability and settling time on encoding of excitatory input**. Simulations like those in Figure 5 were repeated but this time *g*_*inh*_ and *g*_*KCC*2_ were kept constant while *g*_*exc*_ was stepped to randomly chosen values at 10 s intervals. Transmitted information and ionic memory were calculated as shown in Figure 5B but now based on the mutual information between *g*_*exc*_ and ISR. **(A)** For each KCC2 level, we computed transmitted information about *g*_*exc*_ just after the step change in conductance (*black*), 10 s after the step change (*red*) as well as transmitted information under static [Cl^−^]_i_ conditions (*green*). **(B)** The transmitted information is plotted as a function of time elapsed since the last step change in conductance after normalization by the amount of information under static [Cl^−^]_i_ conditions. **(C)** Ionic memory just after the step change in *g*_*exc*_ was computed for various KCC2 levels. **(D)** Ionic memory of excitatory activity is shown as a function of the time elapsed since the last step change in *g*_*exc*_. **(E)** When KCC2 level was reduced, sustained stimulation with weak *g*_*exc*_ triggered spiking whereas shorter stimulations did not. Sample traces show membrane potential (*top*), [Cl^−^]_i_ (*middle*), and ISR (*bottom*). **(F)** Prolonged stimuli of different intensities were applied to the model. Weak stimuli that were never detected by the model with a high KCC2 level were eventually detected by the model with a reduced KCC2 level.

**Figure 7:**
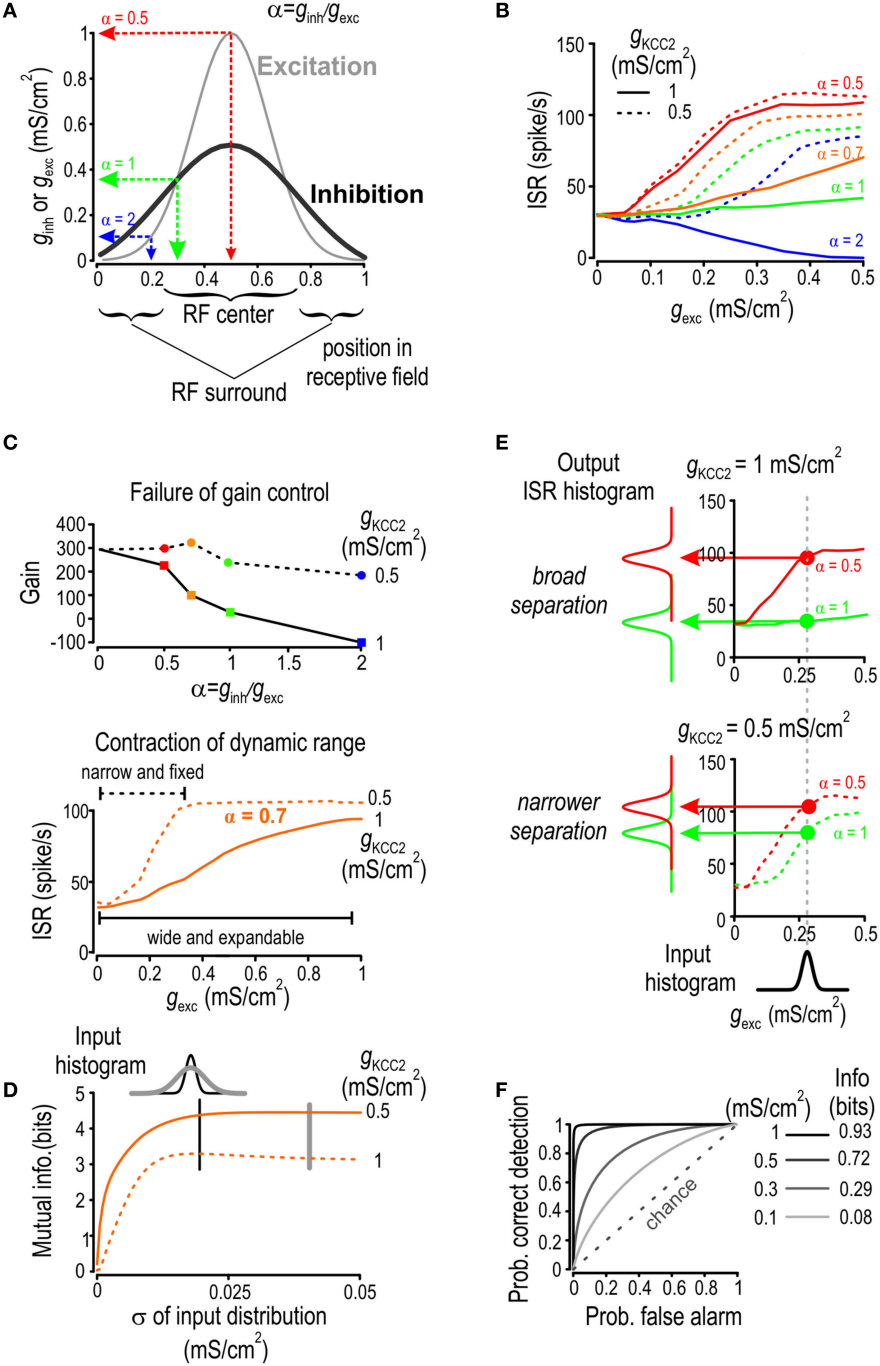
**KCC2 downregulation reduces discriminability**. **(A)** Location within a receptive field (RF) is parameterized by α = *g*_*inh*_/*g*_*exc*_, which is high in the RF periphery and low at the RF center. **(B)** For four different locations in the RF and over a range of stimulus intensities, we computed the average spike rate given a background excitation of 0.5 mS/cm^2^. Both *g*_*inh*_ and *g*_*exc*_ were generated through Ornstein Uhlenbeck processes with different means. **(C)** Gain of the input-output curves from B is shown relative to α. Model with normal KCC2 level (*solid line*) exhibits effective gain modulation whereas the one with reduced KCC2 level (*dotted line*) does not (*top*). The curves obtaiend with α = 0.7 are redrawn (*bottom*) to illustrate how the failure of gain control leads to contraction of the dynamic range. **(D)** For Gaussian distributions of *g*_*exc*_ with different standard deviations but equal means, we computed the level of mutual information between *g*_*exc*_ and ISR for normal and reduced KCC2 level. Mutual information was consistently less for reduced KCC2 level (*dashed* line) compared with normal KCC2 level (*solid* line). **(E)** For a given *g*_*exc*_ distribution, we computed output ISR distributions for α = 0.5 (*red*) or 1 (*green*) for a normal KCC2 level (*top*) and for a reduced KCC2 level (*bottom*). **(F)** Given the output ISR distribution in **(E)** as well as distributions obtained for other KCC2 levels, ROC curves were computed by plotting the probability of correct detection against the probability of false alarm, mutual information between input location and ISR was also computed under the assumption that a priori probability of the input of being at each location is 0.5.

Thus far, we have focused on the encoding and decoding of inhibitory input because of its direct connection with Cl^−^ flux. However, excitatory synaptic input also affects *E*_GABA_, albeit indirectly, since depolarization caused by synaptic excitation contributes to a depolarizing shift in *E*_GABA_ (Figure 1D). As an aside, excitatory and inhibitory input co-vary during realistic stimulation (Seriés et al., 2003; Haider et al., 2006). But putting aside co-variations (until **Figure 7**), *g*_*exc*_ fluctuations could cause Cl^−^ fluctuations even if *g*_*inh*_ remains constant, which led us to hypothesize that Cl^−^ instability could also form an ionic substrate for memory of past *g*_*exc*_ and that it could impair encoding of the present value of *g*_*exc*_. To test this, we repeated the simulations reported in Figure 5 but this time *g*_*inh*_ was kept constant while *g*_*exc*_ underwent intermittent step-changes. We computed transmitted information about *g*_*exc*_ based on ISR immediately and 10 s after the step change in *g*_*inh*_. For comparison, we also calculated transmitted information with [Cl^−^]_i_ held constant at the average value of [Cl^−^]_i_ associated with each KCC2 level (Figure 6A).

Like for encoding of *g*_*inh*_, the information transmitted about *g*_*exc*_ immediately after the step change in conductance decreased for low KCC2 levels. But interestingly, and unexpectedly, compared with static [Cl^−^]_i_ conditions, transmitted information was increased after waiting long enough for [Cl^−^]_i_ to reach steady state. This is best illustrated by plotting transmitted information as a function of time elapsed since the step change in conductance normalized to transmitted information with constant [Cl^−^]_i_ (Figure 6B). Computations confirmed that fluctuations in ionic concentrations create a Cl^−^-based memory of past *g*_*exc*_ (Figures 6C,D) that was much weaker than the memory of past *g*_*inh*_ (cf. Figure 5) but interestingly, could still affect information transfer.

Like for *g*_*inh*_, *g*_*exc*_ modulates ISR via two distinct pathways: its immediate and direct effect via excitatory current, and the slower and indirect effect via [Cl^−^]_i_, where the latter affects the influence of concurrent inhibition on ISR. A critical issue is whether the direct and indirect effects oppose or reinforce one another. In the case of *g*_*inh*_, a large conductance will reduce ISR but the associated shift in *E*_GABA_ will undermine that effect (see Figure 1D). In contrast, in the case of *g*_*exc*_, a large conductance will increase ISR and the associated shift in *E*_GABA_ will enhance that effect (by reducing the efficacy of inhibition), meaning the direct and indirect effects are reinforcing. This may, under certain conditions, be beneficial: for low KCC2 levels, weak excitatory inputs (that would normally fail to elicit spiking) can, if sustained for long enough, cause progressive Cl^−^ accumulation and thus disinhibition to the point where spiking is elicited (Figure 6E). Re-testing with inputs of different durations and strengths reveals that weak but *sustained g*_*exc*_ stimuli that would go undetected under normal conditions can be decoded from the steady-state firing rate when KCC2 is downregulated (Figure 6F). Thus, *detection* of sustained weak stimuli can be enhanced by reduced KCC2 activity, which could be beneficial or deleterious depending on context. For example, this could allow subtle mechanical stimuli such as movement of clothes over the skin to activate nociceptive spinal neurons in neuropathic pain conditions—a deleterious effect—but it may also facilitate the establishment of excitatory synapses during early stages of development—a beneficial effect. Furthermore, the same changes would be detrimental for discrimination of stimuli near the upper end of the dynamic range (see below).

### Impact of subtle Cl^−^ dysregulation on discrimination

The decrease in information transmission studied in Figures 5, 6 provides a generic demonstration of how mild Cl^−^ dysregulation may impair neural coding. To provide physiological context, we also simulated a scenario in which tuning curves for excitation and inhibition define a receptive field (RF): excitation and inhibition are both strongest at the RF center, but because inhibition has a broader tuning curve, the *g*_*inh*_/*g*_*exc*_ ratio is greater at the RF periphery (Figure 7A). In this commonly occurring pattern (Ben-Yishai et al., 1995; Suga, 1997; Seriés et al., 2003), inhibition serves to sharpen the tuning curve for net excitation and, in this way, improves spatial acuity or feature selectivity (Marr, 1982). As a preliminary step to assess the impact of Cl^−^ dysregulation on discrimination, we computed the output firing rate of a model neuron as a function of the stimulus location in its RF as parameterized by the *g*_*inh*_/*g*_*exc*_ ratio for different stimulus strengths and KCC2 levels (Figure 7B).

The input-output curves in Figure 7B exhibit features indicative of deteriorated neural coding such as failure of gain control and contraction of dynamic range. To investigate this more quantitatively, we first computed the gain of the input-output curves for a given stimulus strength (*g*_exc_ = 0.3 mS/cm^2^; Figure 7C) which revealed how increasing the *g*_*inh*_/*g*_*exc*_ ratio leads to a linear decrease in input-output gain for a normal KCC2 level whereas gain control failed when KCC2 level was reduced (Figure 7C *top*). Such a failure prevents inhibition from extending the dynamic range, which effectively leads to contraction of the dynamic range when KCC2 level is reduced (Figure 7C *bottom*). As neurons can efficiently encode stimulus strength only if the stimulus distribution falls within the dynamic range, we hypothesized that contraction of dynamic range caused by Cl^−^ dysregulation would impair encoding of broadly distributed input. This was confirmed by plotting Shannon-Weaver mutual information as a function of the width of the input distribution in scenarios of both efficient and compromised KCC2 activity (Figure 7D). As predicted, contraction of dynamic range caused by mildly reduced KCC2 level led to reduced information about input strength and this degradation worsened in proportion to the width of the input distribution.

Having shown that Cl^−^ dysregulation can impair information about input strength, we hypothesized that it could also impair encoding of stimulus location within the RF since input-output curves corresponding to different stimulus locations get closer to each other when KCC2 level is reduced (see Figure 7B). This implies decreased discriminability. To test this hypothesis, we investigated whether the proportionality constant between *g*_*inh*_ and *g*_*exc*_ could be unambiguously inferred from ISR for scenarios of normal and reduced KCC2 levels (Figure 7E). We submitted the model neuron to inputs whose *g*_*inh*_/*g*_*exc*_ ratio corresponds to stimulation within the center or periphery of the RF and we compared the distributions of the resulting ISR vectors. Whereas, the separation between the two ISR distributions is broad for normal KCC2 levels, it becomes narrower when KCC2 level is reduced. Intuitively, broader separation of ISR distributions enables clearer discrimination of stimulus location. Quantifying this with Shannon-Weaver mutual information requires assumptions about the prior probability of the stimulus location; for illustration sake, we assumed each location to have equal probability. The computed mutual information between the input location and ISR was decreased in conditions of impaired KCC2 activity (Figure 7F *right inset*).

To quantify changes in discriminability using independent methodology, we turned to signal detection theory and receiver-operating-characteristic (ROC) analysis (Marr, 1982; Green and Swets, 1988). For any observed ISR value, one can decide if this value arises from a stimulus within the RF center or periphery based on whether the observed ISR lies above or below a given decision threshold. Determination of the optimal value of the decision threshold is based on the tradeoff between false positive and false negative decisions and does not, therefore, require any assumption about the prior distribution. That said, the quality of discrimination can be assessed through ROC diagrams obtained by varying the decision threshold and then plotting the probability of correct detection against the probability of false alarms (Zweig and Campbell, 1993; Figure 7F). The ROC curve for pure chance is a straight line at 45° and the quality of discrimination increases as the actual ROC curve diverges from this diagonal. The ROC curve obtained with a high KCC2 level indicates near perfect discriminability while these obtained for reduced KCC2 levels tend toward chance levels.

In conclusion, testing the impact of Cl^−^ dysregulation in the context of a receptive field showed, in a physiologically relevant paradigm, how modest KCC2 hypofunction disrupts neural coding. This together with other metrics (e.g., reduced mutual information) and the observed disruption of processes like gain control all demonstrate the degradation of neural coding that occurs when Cl^−^ regulation is compromised.

## Discussion

The importance of Cl^−^ regulation for fast synaptic inhibition is well established (Thompson and Gahwiler, 1989; Staley et al., 1995; DeFazio et al., 2000; Coull et al., 2003; Jin et al., 2005; Huberfeld et al., 2007; Blaesse et al., 2009; Hewitt et al., 2009; Boulenguez et al., 2010). Disinhibition caused by downregulation of KCC2 occurs in several pathological conditions (De Koninck, 2007; Kahle et al., 2008; Kaila and Miles, 2010). Sufficient downregulation of KCC2 results in a gross depolarizing shift in *E*_GABA_, which in turn causes reduction (and potentially even inversion) of the normally outward Cl^−^ current through GABA_A_ channels. An important additional consideration is that modest reductions in KCC2 may go undetected if one were to test affected neurons with only weak Cl^−^ loads. Yet strong Cl^−^ loads are routinely experienced by neurons in the intact brain and can overwhelm Cl^−^ extrusion capacity, especially in certain subcellular compartments like dendrites (Doyon et al., 2011). Thus, even a modest reduction of KCC2 can be consequential for neural coding that involves variation in GABAergic input, including situations in which GABAergic and glutamatergic input co-vary. From this starting point, the current study set out to quantify how KCC2 level impacts the ability of a neuron to handle realistically fluctuating Cl^−^ loads and what consequences this has on neural coding.

We found that even modest reduction of KCC2 allowed Cl^−^ extrusion capacity to be more easily overwhelmed by transient Cl^−^ loads resulting in increased lability of [Cl^−^]_i_. But not only did [Cl^−^]_i_ undergo larger changes, it was also slower to stabilize at the new steady-state values, i.e., Cl^−^ regulation was weaker *and* slower. Our simulations also showed that the SAV ratio as well as the strength and frequency of input fluctuations had important effects. The increase in Cl^−^ lability and settling time that was observed after even subtle reduction of KCC2 level had deleterious consequences on neural coding. These consequences depend on a multitude of interacting factors that are difficult to isolate and control for experimentally, hence the utility of computer simulations that allow for more rigorous quantification while avoiding uncontrolled factors. Indeed, our simulations clearly show that increased Cl^−^ lability and settling time, even in response to modest KCC2 downregulation, reduced the information carried by the ISR about GABAergic and/or glutamatergic input.

But while increased Cl^−^ dynamics were generally found to counterproductive, the specific effects are context dependent. Indeed, we found in certain circumstances that mild KCC2 downregulation resulted in increased information transfer for excitatory signals. This could certainly benefit the detection of weak excitatory signals, which may play a beneficial role during development, but so too could it compromise discrimination under other conditions. This last observation makes the point that Cl^−^ dynamics are not good or bad in their own right, but generally speaking, it is counterproductive for inhibition to weaken (because of impaired Cl^−^ regulation) every time inhibition is called upon. To illustrate, consider that if adaptation—reduced excitation during sustained stimulation—is generally beneficial (Wark et al., 2007), then the phenomenon we describe—reduced inhibition during sustained stimulation—is liable to be detrimental under the same circumstances.

**Figure 8:**
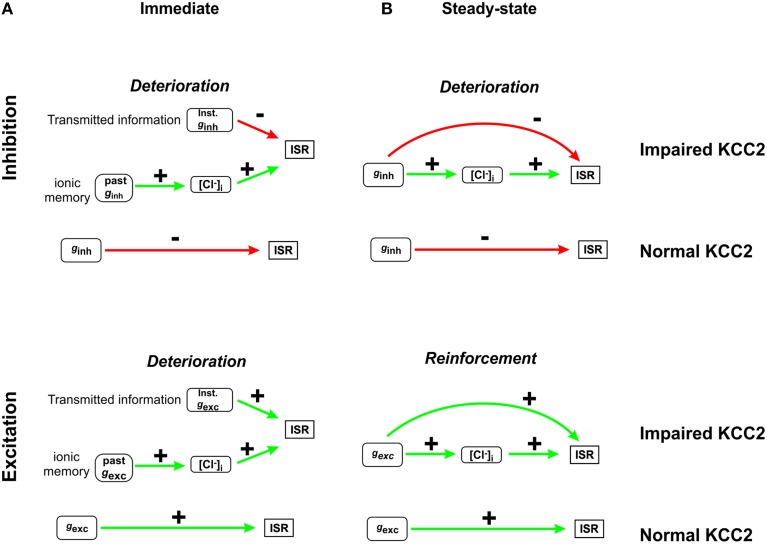
**Summary of how Cl^−^ instability affects neural coding**. **(A)** Immediately after a step change in *g*_*inh*_, ISR is determined by two factors: the present value of *g*_*inh*_ and [Cl^−^]_i_, which is a function of past *g*_*inh*_ (*top*). The dependence on past *g*_*inh*_ constitutes ionic memory. Slow Cl^−^ regulation prolongs ionic memory, which invariably reduces transmitted information. A similar situation arises right after a step change in *g*_*exc*_ (*bottom*). **(B)** Long after a step change in *g*_*inh*_ (after [Cl^−^]_i_ converges to its new steady state value), ISR becomes independent of past *g*_*inh*_ but the present value of *g*_*inh*_ affects ISR in two different ways: through the direct effects of *g*_*inh*_ as well as through the indirect effects of *g*_*inh*_ via [Cl^−^]_i_ determination. When the direct and indirect effects oppose one another, as in the case of inhibitory inputs (*top*), the dynamic range of ISR is compressed and transmitted information is reduced. Conversely, when the direct and indirect effects reinforce one another, as in the case of excitatory inputs (*bottom*), and the range of detected stimuli is increased.

Figure 8 summarizes the two mechanisms through which Cl^−^ instability affects information transfer. On short time scales (e.g., shortly after a step change in *g*_*inh*_), two factors compete to determine ISR: the present value of *g*_*inh*_ (or *g*_*exc*_) and the present value of [Cl^−^]_i_, which is determined by past values of *g*_*inh*_ (or *g*_*exc*_). Since both factors affect ISR, the latter is prevented from faithfully reflecting information about present input (Figure 8A). On longer time scales (e.g., long after a step change in input), ionic memory wanes as [Cl^−^]_i_ converges toward its steady-state, but that steady-state is still non-static, which has opposite effects on the encoding of excitatory and inhibitory inputs (Figure 8B). In summary, KCC2 hypofunction results in Cl^−^ regulation becoming weak (which results in lability of steady-state [Cl^−^]_i_) and slow (which results in ionic memory and long settling times). The former effect can improve or degrade the encoding of input signals, depending on the nature (inhibitory or excitatory) of the input, whereas the latter effect invariably degrades the encoding of input signals. Yet, in the context of stimulus localization or feature selectivity (i.e., discrimination based on receptive field separation), the signal normally comprises a combination of inhibitory and excitatory inputs; thus, it is unclear *a priori* how the above described mechanisms will combine to affect neural coding. Our simulations demonstrate that mild Cl^−^ dysregulation prevents efficient discrimination of stimulus strength and position. Thus, the net effect of mechanisms summarized in Figure 8 is to degrade neural coding under typical stimulus conditions.

Drawing a link between clinical symptoms and mild Cl^−^ dysregulation is not obvious but, in that regard, our study makes several unintuitive predictions that may help in future testing or in the re-evaluation of available data. First, a compensatory increase in GABA_A_ input that mitigates the impact of gross changes in *E*_GABA_ on inhibition will paradoxically exaggerate Cl^−^ fluctuations. Thus, therapies aimed at enhancing residual inhibitory function may, in some circumstance, exacerbate rather than mitigate Cl^−^ fluctuations, causing clinical symptoms to worsen rather than improve, or causing the occurrence of debilitating side effects. Benzodiazepines have been shown to have paradoxical effects in neonatal animals (Koch et al., 2008) and the clinical literature is replete with examples of such effects (Bäckström et al., 2011; Bruining et al., 2015). Indeed, elevated intracellular Cl^−^ is thought to compromise the anticonvulsant effects of benzodiazepines and barbiturates (Dzhala et al., 2005) consistent with relatively low efficacy of those drugs in neonatal seizures (Painter et al., 1999); moreover, barbiturates may reduce seizure symptoms without normalizing electroencephalographic activity (Scher et al., 2003), implying that network hyperactivity is unabated, and could in fact be perpetuated by paradoxically excitatory inhibition (Ellender et al., 2014; but see also Fröhlich et al., 2005). In contrast, therapies that restore Cl^−^ extrusion capacity (Gagnon et al., 2013) may convey clinical benefits even before Cl^−^ dysregulation has progressed far enough to cause gross disinhibition. With normal KCC2 levels already providing for excess extrusion capacity in healthy central neurons, enhancing KCC2 is not expected to cause excess inhibition (Doyon et al., 2011; Gagnon et al., 2013). Differential expression of various KCC2 transcripts in schizophrenia and affective disorders (Tao et al., 2012) and an “immature” ratio of NKCC1 to KCC2 in schizophrenia (Hyde et al., 2011; Kalkman, 2011) are both suggestive of clinically meaningful link between those diseases and Cl^−^ dysregulation. Importantly, our results suggest that disinhibition need not cause frank hyperexcitability (e.g., seizures) in order to be pathologically relevant; on the contrary, subtle Cl^−^ dysregulation compromises neural coding and such changes will invariably precede the development of gross hyperexcitability as KCC2 is progressively downregulated. This suggests that sensory, cognitive and motor function could be disrupted by inconspicuous Cl^−^ dysregulation, meaning that even modest KCC2 hypofunction should be cause for concern and, in turn, the remediation of modest KCC2 hypofunction may be clinically beneficial.

## Author contributions

All the authors contributed to the conception of the study. ND developed the model and performed the analysis. All authors contributed to the writing of the manuscript.

## Funding

The work was funded by grants from the Natural Sciences and Engineering Research Council of Canada to ND, YDK, and SAP and from the Fonds Québécois de la Recherche sur la Nature et les Technologies to ND. It was also supported by a Canadian Institutes of Health Research New Investigator Award and an Ontario Early Researcher Award to SAP and a Canada Research Chair to YDK.

### Conflict of interest statement

The authors declare that the research was conducted in the absence of any commercial or financial relationships that could be construed as a potential conflict of interest. The reviewer Michael E. Hildebrand and handling Editor Gerald W. Zamponi declared a current/past collaboration and the handling Editor states that the process nevertheless met the standards of a fair and objective review.

## Supplementary material

Supplementary Figure S1 **Impact of input frequency on [Cl^−^]_i_ fluctuations**. For various frequency of sinusoidal inhibitory input (0.1, 0.5, and 1 Hz) we monitored the model neuron response in terms of ISR **(A)** and [Cl^−^]_i_ **(B)**. The branches of the ISR input are wider apart for low frequency input while the elliptical [Cl^−^]_i_ response is wider for lower input frequency. **(C)** For the simulations performed in **(A,B)**, ISR is replotted as a function of [Cl^−^]_i_ which reveals slightly slanted ellipses resembling more a vertical line as input frequency is increased.

Supplementary Figure S2 **Determination of parameters for compensated inhibition and static [Cl^−^]_i_ simulations for simulations shown in Figure 5. (A)** For a value of *g*_*exc*_ leading to an ISR = 80 Hz in the absence of inhibition, we identified pairs of *g*_*KCC*2_-*g*_*inh*_ leading to an ISR reduction to 30 Hz**. (B)** The value of [Cl^−^]_i_ corresponding to each scenario identified in **(A)** is plotted as a function of KCC2 activity. For different KCC2 levels, we ran simulations in which *g*_*KCC*2_ and *g*_*exc*_ were kept constant while *g*_*inh*_ was kept constant over 10 s intervals and changed in a step manner to a new value chosen randomly from a Gaussian distribution with mean identified in **(A)**.
