# Supplementary figures and images for: Mild KCC2 Hypofunction Causes Inconspicuous Chloride Dysregulation that Degrades Neural Coding
Source: Front Cell Neurosci. 2016 Jan 29;9:516. doi: 10.3389/fncel.2015.00516 (PMC4731508; doi:10.3389/fncel.2015.00516)

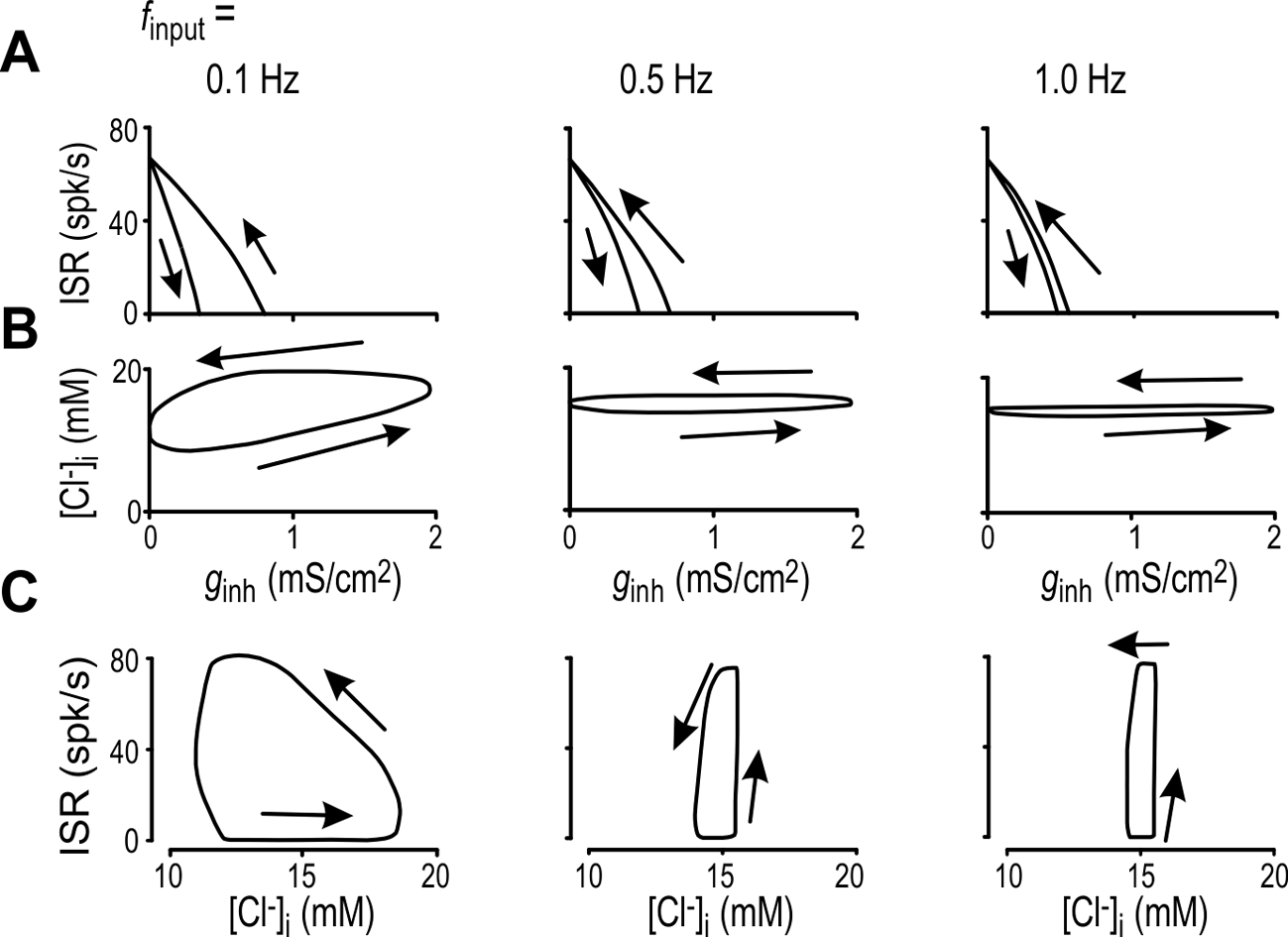

Supplement: Supplementary Figure S1 — Impact of input frequency on [Cl−]i fluctuations. For various frequency of sinusoidal inhibitory input (0.1, 0.5, and 1 Hz) we monitored the model neuron response in terms of ISR (A) and [Cl−]i (B). The branches of the ISR input are wider apart for low frequency input while the elliptical [Cl−]i response is wider for lower input frequency. (C) For the simulations performed in (A,B), ISR is replotted as a function of [Cl−]i which reveals slightly slanted ellipses resembling more a vertical line as input frequency is increased. [file Image1.TIF]

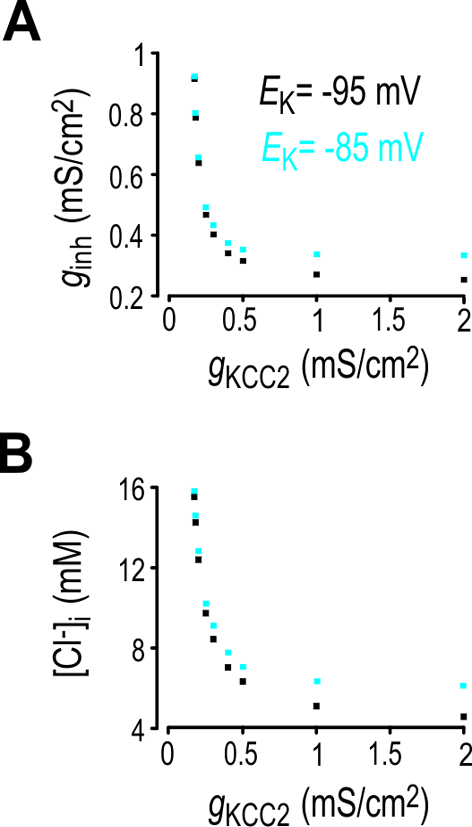

Supplement: Supplementary Figure S2 — Determination of parameters for compensated inhibition and static [Cl−]i simulations for simulations shown in Figure 5. (A) For a value of gexc leading to an ISR = 80 Hz in the absence of inhibition, we identified pairs of gKCC2-ginh leading to an ISR reduction to 30 Hz. (B) The value of [Cl−]i corresponding to each scenario identified in (A) is plotted as a function of KCC2 activity. For different KCC2 levels, we ran simulations in which gKCC2 and gexc were kept constant while ginh was kept constant over 10 s intervals and changed in a step manner to a new value chosen randomly from a Gaussian distribution with mean identified in (A). [file Image2.TIF]
